# Breaking the resistance barrier: synergistic evolution of CAR-T cells and bispecific antibodies in the era of precision immuno-oncology

**DOI:** 10.3389/fimmu.2026.1859167

**Published:** 2026-06-30

**Authors:** Xiaoqiang Wang, Yali Liu, Qiang Yang, Hai Zhao

**Affiliations:** 1 Department of Neurosurgery, Lanzhou University Second Hospital, Lanzhou, Gansu, China; 2 Department of Neurosurgery, The Affiliated Hospital of Qingdao University, Qingdao, Shandong, China

**Keywords:** BsAbs, cancer immunotherapy, combination strategies, next-generation CAR-T cells, solid tumors, treatment sequencing

## Abstract

The therapeutic landscape of oncology has undergone a profound paradigm shift, transitioning from conventional cytotoxic regimens to a sophisticated era of precision immunotherapy. Despite the remarkable clinical success of immune checkpoint inhibitors, significant challenges such as primary resistance, limited T-cell infiltration in solid tumors, and severe immune-related adverse events persist. As the second volume of “The Role of Immunotherapy in Cancer Therapy and Its Challenges” Community Series, this review systematically evaluates the recent breakthroughs and persistent hurdles in CAR-T cell therapy and bispecific antibodies (BsAbs). We emphasize a critical strategic shift: transitioning these potent modalities from late-stage salvage therapies to earlier treatment lines to preserve the patient’s immune repertoire and improve long-term survival. Furthermore, we dissect the molecular engineering of innovative CAR-T and BsAb constructs—such as armored CARs and multi-specific engagers—specifically designed to antagonize the immunosuppressive tumor microenvironment (TME) in solid cancers. A central focus is placed on the optimization of combination strategies, including the synergistic integration of cellular therapies with hematopoietic stem cell transplantation (HSCT) and targeted agents to eradicate minimal residual disease (MRD). By synthesizing the latest clinical data on overall survival (OS) and progression-free survival (PFS), we propose an evidence-based framework for sequential therapy and toxicity management. Ultimately, this review aims to provide a roadmap for the next generation of personalized immuno-oncology, addressing how innovative molecular design and strategic timing can overcome current resistance barriers and redefine the standard of care for refractory malignancies.

## Introduction

The past decade has witnessed an unprecedented acceleration in the development of cancer therapeutics, moving away from the “one-size-fits-all” approach of cytotoxic chemotherapy toward the nuanced realms of precision and personalized medicine (1–3). At the heart of this revolution lies immunotherapy, which has fundamentally redefined the prognosis for patients with previously untreatable malignancies. However, as we embark on the second volume of this Research Topic, the initial euphoria surrounding early breakthroughs is being met with the sober reality of clinical resistance and the unique biological barriers presented by solid tumors (4, 5). Currently, the field stands at a critical juncture where the primary objective is no longer just “activation” of the immune system, but the sophisticated “optimization” of its effector functions.

**Figure 1:**
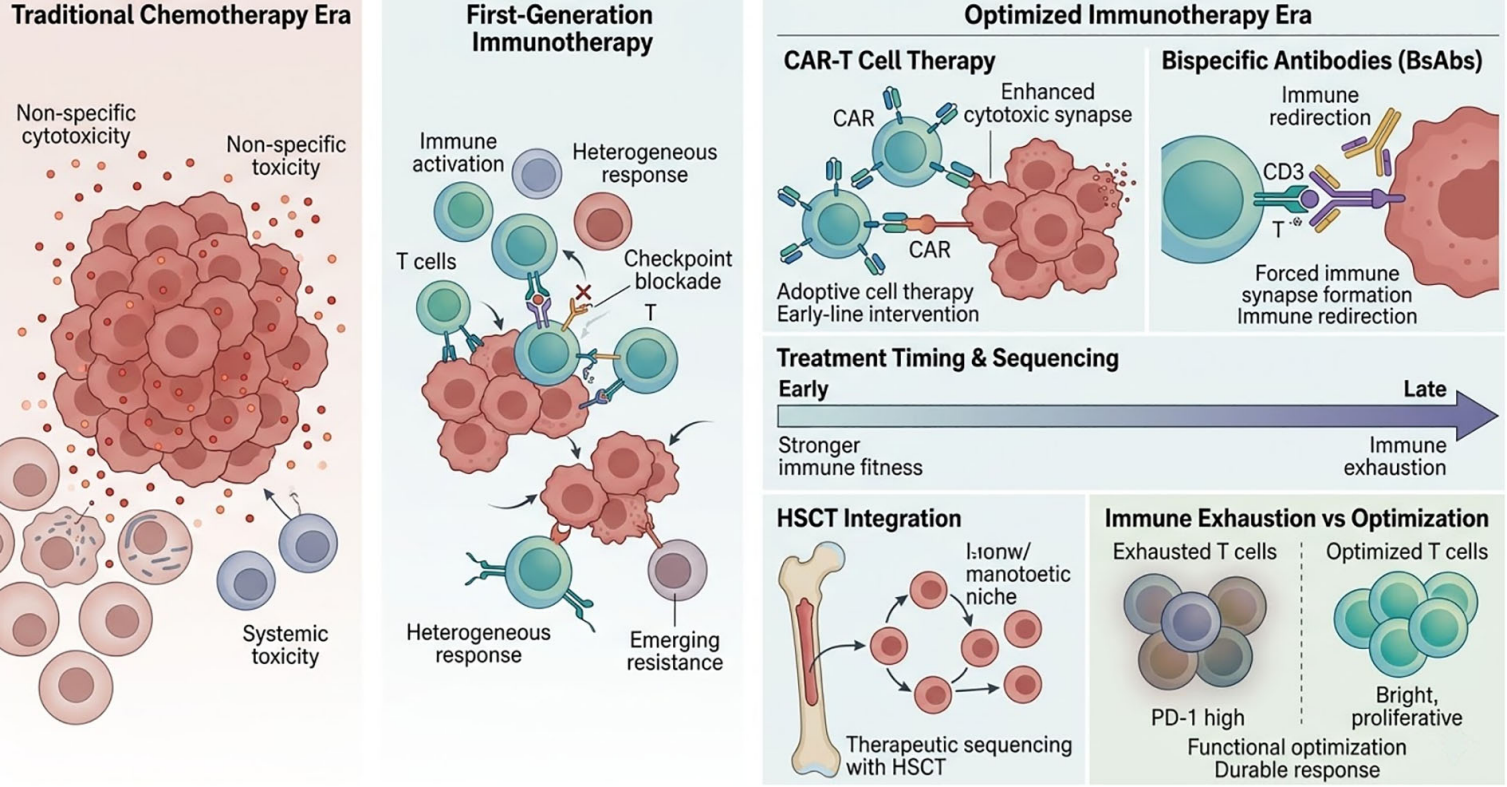
The evolutionary paradigm of cancer therapeutics from non-specific cytotoxicity to precision immuno-oncology. The schematic delineates the conceptual progression of oncological treatments across three distinct therapeutic eras. The left panel illustrates the traditional chemotherapy era, characterized by the deployment of non-specific cytotoxic agents that induce widespread collateral damage to healthy tissues and systemic toxicity, alongside profound immune suppression. The center panel depicts first-generation immunotherapy, highlighting the initial activation of the host immune system via immune checkpoint blockade. While this approach facilitates T-cell engagement with malignant cells, it frequently results in a heterogeneous clinical response and the subsequent emergence of resistant tumor clones. The right panel details the contemporary era of optimized immunotherapy, which emphasizes functional cellular engineering and strategic treatment sequencing. Key components include adoptive cell therapy utilizing CAR-T cells to form enhanced cytotoxic synapses for robust tumor eradication, and the application of BsAbs is to forcibly redirect CD3-positive T cells to tumor-associated antigens via immune synapse formation. Furthermore, the schematic emphasizes the critical importance of treatment timing, contrasting the robust immune fitness preserved through early-line intervention against the profound T-cell exhaustion, marked by high PD-1 expression, observed in late-line salvage settings. The strategic integration of these advanced immunotherapeutic modalities within the HSCT transplant niche is depicted as a synergistic approach to overcome functional exhaustion and secure durable clinical remission. ASCT, autologous stem cell transplantation; BsAbs, bispecific antibodies; CAR-T, chimeric antigen receptor T-cell; EFS, event-free survival; HR, hazard ratio; IVIG, intravenous immunoglobulin; LBCL, large B-cell lymphoma; PFS, progression-free survival; $T_N$, naive T-cell; $T_{SCM}$, stem cell memory T-cell.

Two pillars of this modern arsenal—CAR-T cell therapy and BsAbs—have demonstrated extraordinary efficacy in hematological cancers (6–8). Yet, their integration into the broader oncological framework remains complex. A pivotal question facing clinicians and researchers is the timing of intervention; evidence increasingly suggests that deploying these potent agents in earlier lines of treatment may prevent the cumulative immune exhaustion associated with multiple rounds of chemotherapy, thereby maximizing therapeutic durability. Furthermore, the interplay between adoptive cell therapies and established procedures like HSCT requires meticulous sequencing to achieve deep, molecular remissions while minimizing overlapping toxicities (**Figure 1**).

The challenge is further magnified in the context of solid tumors, where the physical stroma and an immunosuppressive tumor microenvironment (TME) act as formidable bastions against immune infiltration (9). To penetrate these defenses, the next generation of immunotherapy relies on innovative molecular engineering—creating “armored” chimeric antigen receptor T cell (CAR-T therapy) capable of remodeling the TME and bispecific molecules with enhanced specificity and reduced off-target effects (10). However, increasing the potency of these “living drugs” inevitably heightens the risk of cytokine release syndrome (CRS) and neurotoxicity, necessitating a delicate balance between aggressive eradication and patient safety (11, 12). This review provides a comprehensive analysis of these shifting paradigms, exploring how the synergy of molecular innovation and strategic therapeutic sequencing can overcome current limitations. By addressing the optimization of combination therapies and the specific hurdles of solid tumor treatment, we aim to delineate the future trajectory of immunotherapy in the quest for sustained, curative outcomes.

To ensure a rigorous and comprehensive synthesis of the literature, a systematic search was performed across PubMed, Embase, and Google Scholar database platforms up to May 2026. Search algorithms utilized combinations of medical subject headings (MeSH) and high-yield keywords, including: ‘CAR-T cell therapy’, ‘BsAbs’, ‘solid tumor microenvironment’, ‘treatment sequencing’, ‘epitope spreading’, and ‘precision immuno-oncology’. The screening strategy relied on peer-reviewed, English-language primary research articles, landmark clinical trial reports (Phases I–III), and high-impact mechanistic studies. Priority was systematically allocated to recent literature (2022–2026) to reflect the contemporary translational and molecular landscape of cancer immunotherapies.

## Optimization of CAR-T and bispecifics in early-line treatment

The shift of CAR-T cell therapy and BsAbs from “salvage” therapies to earlier lines of intervention is driven by the biological imperative of preserving T-cell fitness and preventing the clonal evolution of resistant malignancies. In late-line settings, the patient’s T-cell compartment is often profoundly senescent due to cumulative exposure to lymphotoxic agents like fludarabine or alkylators. By moving these therapies to the second or even first line, we can harvest “healthier” autologous T-cells with higher proportions of naive (T_N_) and stem cell memory (T_SCM_) phenotypes. These subsets are characterized by superior metabolic plasticity and proliferative potential (13). Translational clinical evidence confirms that a higher baseline frequency of these younger T-cell phenotypes in the apheresis product strongly correlates with enhanced, robust *in vivo* cellular expansion and prolonged progression-free survival (PFS) in patients, whereas cumulative chemotherapy-induced lineage senescence consistently limits therapeutic durability (14). Clinical evidence from landmark trials, such as ZUMA-7 and TRANSFORM, has already challenged the long-standing paradigm of high-dose chemotherapy followed by autologous stem cell transplant (ASCT) for second-line large B-cell lymphoma (15, 16)(**Table 1**).

**Table 1:** Structural architecture, mechanistic attributes, and translational status of next-generation CAR-T and bispecific antibody constructs.

Modality & platform	Molecular / intracellular architecture	Costimulatory & signaling cascade	Key representative trials / clinical targets	Primary resistance & operational limitations	Development / translational stage
Conventional 2nd-Gen CAR-T	Extracellular scFv + CD28 or 4-1BB + intracellular CD3ζ chain	LCK-mediated phosphorylation; activates PI3K/AKT or TRAF downstream pathways	Axicabtagene Ciloleucel (ZUMA-7), Tisagenlecleucel (NCT03575351) / CD19, BCMA	High susceptibility to antigen loss/escape; rapid induction of terminal exhaustion in solid tumor microenvironments	FDA Approved / Standard of Care for hematological malignancies
Armored 4th-Gen CAR-T (TRUCKs)	Conventional 2nd-Gen backbone + antigen-inducible transgenic cytokine cassette (e.g., IL-12, IL-18)	Localized, antigen-dependent cytokine secretion; induction of autocrine/paracrine loops	Meso-IL12 CAR-T, GPC3-IL18 CAR-T / Solid tumors (Mesothelin, GPC3)	Risk of leaky systemic cytokine release syndrome (CRS); requirement for stringent temporal control of transgene translation	Phase I/II Clinical Trials; demonstrating prominent epitope spreading
Fifth-Generation CAR-T (Cytokine-Fused)	Extracellular scFv + CD28/4-1BB + CD3ζ + truncated intracellular domain of IL-2Rβ	Engineered convergence of TCR-like and antigen-dependent JAK/STAT3/5 signaling	Investigational constructs targeting solid tumors / EGFRvIII, Claudin 18.2	Long-term cell-autonomous persistence dynamics require further human validation; fluid nomenclature consensus in the field	Preclinical / Early-Phase I Trials; promotes a robust T<sub>SCM</sub> memory phenotype
Logic-Gated synNotch Circuit	Dual-receptor system: Primary synNotch receptor + secondary inducible canonical CAR construct	Intramembrane proteolytic cleavage; releases synthetic transcription factor (TF)	Multi-targeted GBM trials / EGFRvIII, IL-13Rα2, HER2	Irreversible genomic integration risks; potential host immunogenicity against foreign synthetic TFs; insertional mutagenesis	Preclinical Bench Stage; configured to overcome extreme solid tumor antigen heterogeneity

Clinical evidence from landmark trials, such as ZUMA-7 and TRANSFORM, has already challenged the long-standing paradigm of high-dose chemotherapy followed by ASCT for second-line large B-cell lymphoma. In the ZUMA-7 trial, axicabtagene ciloleucel demonstrated overwhelming superiority over standard second-line salvage-and-transplant care, generating a median event-free survival (EFS) of 8.3 months versus 2.0 months in the control arm, and a significant 60% reduction in the risk of EFS events (17). Similarly, the TRANSFORM study reported a highly significant elongation of median EFS favoring the early CAR-T arm (10.1 months vs. 2.3 months; HR: 0.35), establishing that early cellular deployment significantly thwarts clonal evolution before the tumor acquires resistant sub-clonal variants (18). The underlying advantage lies in the ability to achieve deep molecular remission before the tumor acquires additional driver mutations or complex karyotypes that facilitate antigen escape (19).For BsAbs, the optimization in earlier lines focuses on their “off-the-shelf” availability and lower entry barrier compared to the logistical complexities of CAR-T manufacturing. For BsAbs, the optimization in earlier lines focuses on their ‘off-the-shelf’ availability and lower entry barrier compared to the logistical complexities of CAR-T manufacturing. Representative approved agents, such as the CD19-directed T-cell engager blinatumomab or the BCMA-targeted teclistamab, have redefined early consolidation paradigms (20). In clinical oncology, these constructs are increasingly deployed for rapid, immediate tumor debulking in patients with volatile, high-burden disease to interrupt acute clinical deterioration while alternative cellular therapies are manufactured. Furthermore, the clinical application of BsAbs in earlier lines of treatment facilitates defined, ‘fixed-duration’ therapeutic cycles (e.g., restricted to 4–6 cycles in consolidation phases), which can induce deep, durable clinical remissions while allowing the host immune repertoire to recover from active drug exposure (21). Furthermore, the clinical application of BsAbs in earlier lines of treatment facilitates defined, ‘fixed-duration’ therapeutic cycles, which can induce deep, durable clinical remissions (22). However, this clinical attribute must be carefully contextualized to avoid misleading generalizations regarding toxicity profiles (23). Bispecific T-cell engagers targeting lineage-specific antigens like CD19 regularly produce profound, acute B-cell aplasia during active administration phases, and prolonged exposure frequently culminates in severe hypogammaglobulinemia (24). Crucially, the biological distinction between bispecific molecules and adoptive cellular therapies resides entirely within their pharmacokinetic clearance and *in vivo* persistence, rather than an inherent absence of on-target, off-tumor B-cell toxicities (25). The utilization of fixed-duration BsAb regimens does not bypass lineage depletion during the active phase, but rather relies on the decay of the antibody pool to permit the organic, long-term hematopoietic reconstitution of the endogenous B-cell compartment post-treatment. This is particularly relevant for elderly patients or those with pre-existing comorbidities who may not tolerate the intensive lymphodepletion required for cellular therapies (26).

**Figure 2:**
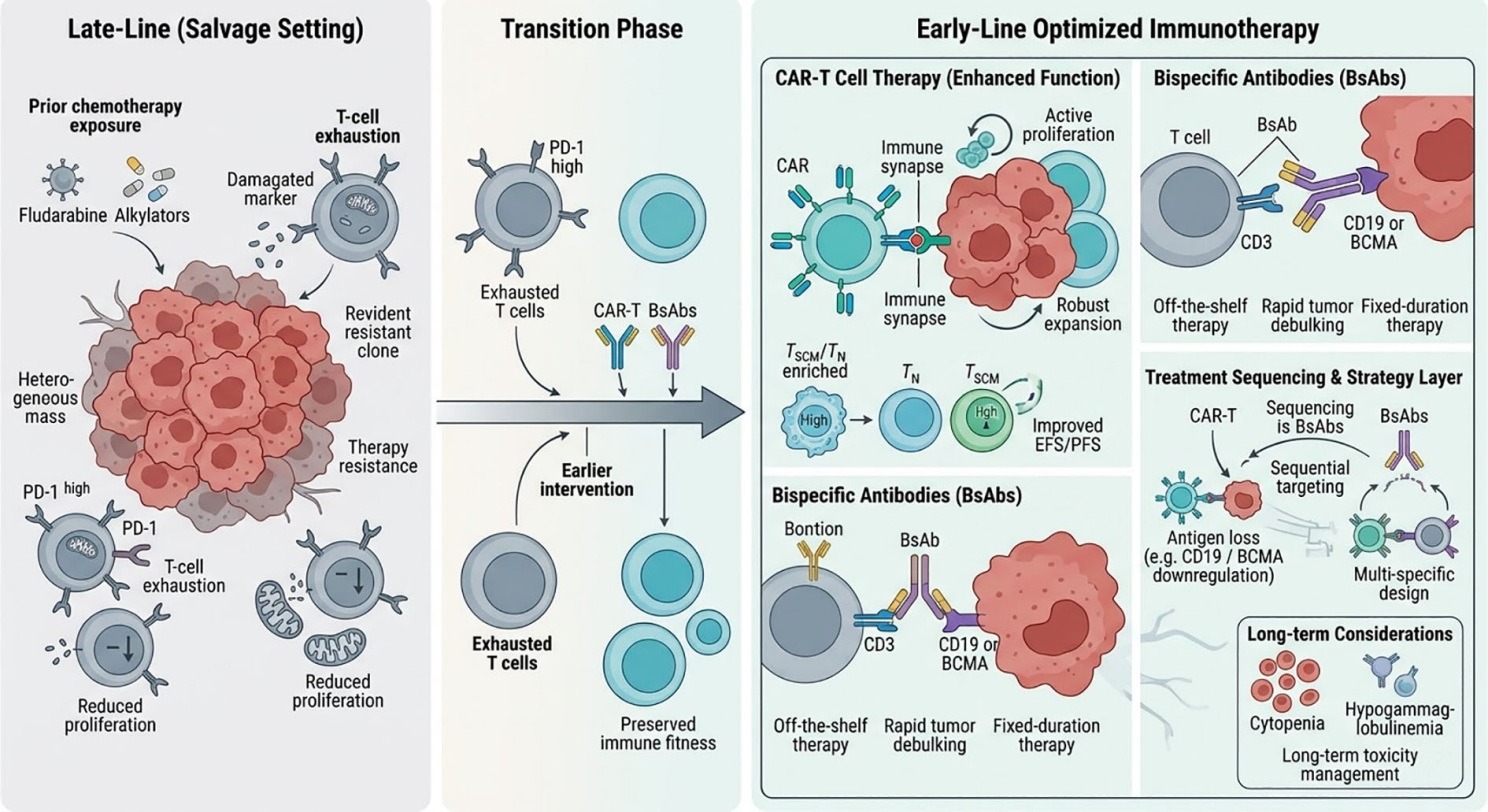
The therapeutic paradigm shift from late-line salvage to early-line precision immunotherapy. The schematic illustrates the rationale and mechanistic advantages of advancing CAR-T cell therapy and BsAbs into earlier oncological treatment lines. The left panel depicts the immunologically compromised microenvironment typical of late-line salvage settings, wherein cumulative exposure to lymphotoxic chemotherapy induces severe T-cell exhaustion. This state is characterized by high PD-1 expression, mitochondrial damage, and reduced proliferative capacity, which cumulatively facilitate clonal evolution and therapy resistance. The central transition phase highlights the fundamental biological advantage of earlier clinical intervention: the preservation of intrinsic immune fitness through the utilization of a robust, non-exhausted T-cell repertoire. The right panel details the optimized clinical dynamics of early-line therapy. Enhanced CAR-T cell function is demonstrated by the formation of robust immunological synapses and active proliferation, specifically leveraging T_SCM_ and T_N_ phenotypes to achieve durable responses and improved progression-free survival. Concurrently, BsAbs are depicted providing rapid, off-the-shelf tumor debulking by physically bridging CD3 on endogenous T cells with specific tumor-associated antigens. The schematic further outlines critical strategic considerations in therapeutic sequencing, emphasizing the risk of target antigen downregulation and the requisite deployment of multi-specific construct designs, alongside the imperative clinical requirement to monitor and manage long-term toxicities including hypogammaglobulinemia and prolonged cytopenias.

However, earlier deployment necessitates a sophisticated understanding of treatment sequencing. However, earlier deployment necessitates a sophisticated understanding of treatment sequencing (27). A major clinical hurdle is the potential for acute antigen escape or the down-regulation of target epitopes early in the disease course, which could permanently restrict future therapeutic selections. For example, the emergence of CD19-negative malignant clones after initial CAR-T exposure, or the prompt down-regulation of BCMA surface densities following front-line bispecific treatments, represents a critical evolutionary dead-end (28). Optimization therefore requires the development of multi-specific agents or the sequential use of non-overlapping targets to ensure that moving a potent therapy forward does not result in a therapeutic dead-end. Managing the long-term toxicity profile also becomes paramount as we treat patients with significantly longer life expectancies. Late-onset, prolonged cytopenias and profound secondary hypogammaglobulinemia are frequently under-addressed in late salvage settings but present severe management risks in early-line patients. Prolonged hypogammaglobulinemia directly translates to an elevated, chronic susceptibility to recurrent, life-threatening sinopulmonary pyogenic infections. This necessitates structured, long-term clinical monitoring and proactive intravenous immunoglobulin replacement therapy to preserve host humoral protection without undermining anti-tumor efficacys (29, 30) (**Figure 2**).

## Synergistic combination and sequential strategies

The integration of CAR-T therapy and BsAbs into multimodal treatment paradigms represents a critical frontier in overcoming primary and adaptive resistance mechanisms. As single-agent efficacies plateau, particularly in high-burden or refractory diseases, synergistic combination and sequential strategies are essential to optimize overall survival and progression-free survival. A cornerstone of this approach is the strategic sequencing with hematopoietic stem cell transplantation. Adoptive cellular therapies and bispecific engagers are increasingly utilized as potent bridging tools to achieve deep MRD negativity. However, their tactical deployment diverges significantly between allogeneic and autologous settings: in ASCT, immunotherapies are primarily used post-transplant as consolidation to eradicate high-risk minimal residual clones within the context of a resetting, lymphodepleted host environment. Conversely, in the allogeneic -HSCT niche, these agents serve as an ultra-potent bridging matrix to force deep pre-transplant MRD negativity, thereby drastically reducing post-transplant relapse rates, or are utilized post-transplant to treat molecular recurrence by harnessing graft-versus-tumor effects without triggering exacerbations of GVHD (31). Conversely, they serve as crucial consolidation or salvage strategies post-transplantation, capitalizing on the lymphodepleted, immunologically resetting environment to enhance donor or autologous T-cell expansion and persistence.

**Figure 3:**
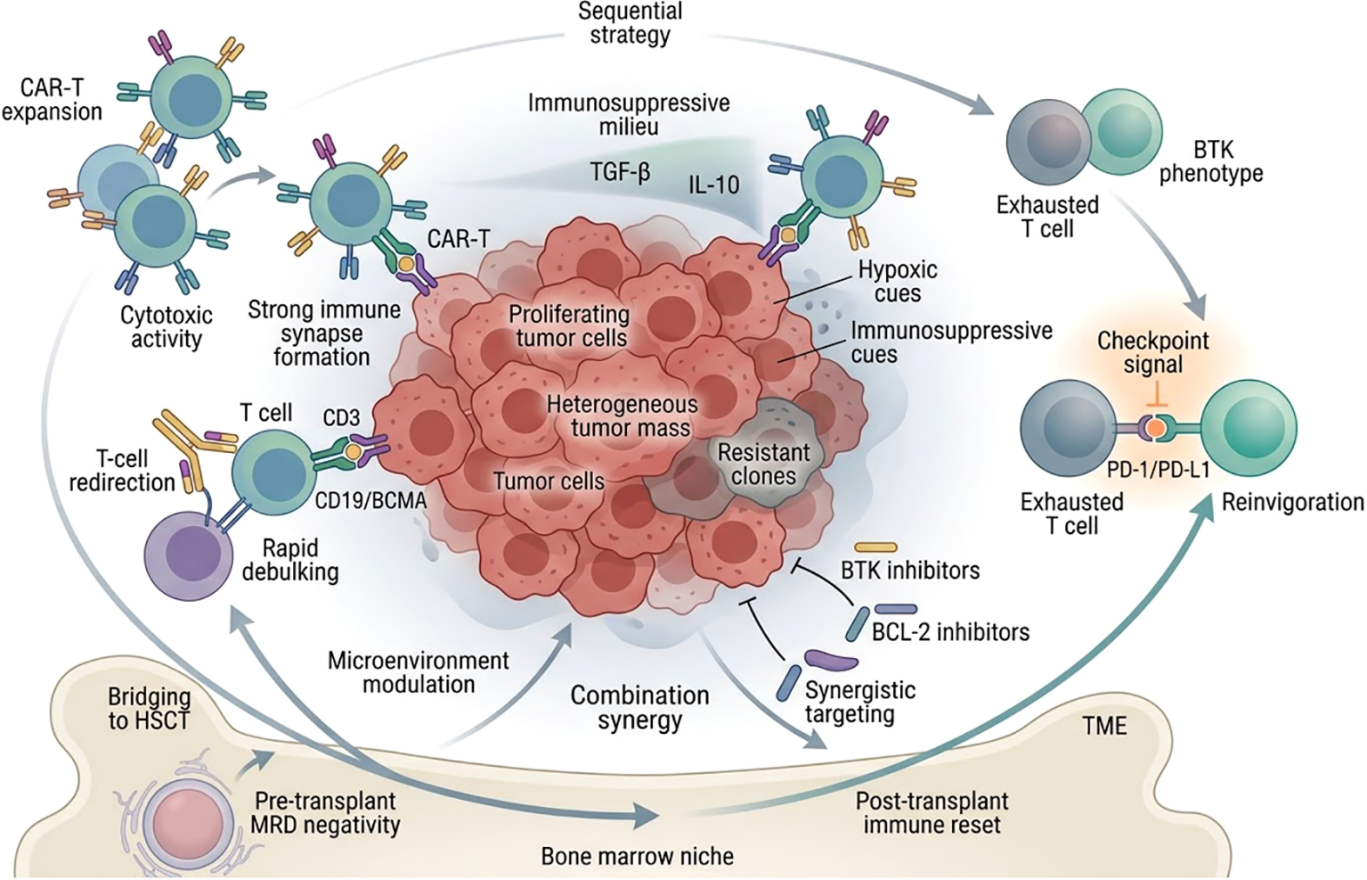
Integrated multimodal strategy for a durable anti-tumor response. The illustration visualizes a unified, spatially integrated landscape of a heterogeneous tumor microenvironment (TME) targeted by a coordinated network of synergistic therapies. A central heterogeneous tumor mass, containing proliferating tumor cells and resistant clones, is surrounded by an immunosuppressive milieu characterized by TGF-β and Interleukin-(IL-)10 gradients, alongside hypoxic and other immunosuppressive cues within the TME. To the left, engineered CAR-T cells undergo clonal expansion and display cytotoxic activity, forming a strong immune synapse, while BsAbs physically link T cells (e.g., CD3) and tumor antigens (such as CD19 or BCMA) for rapid tumor debulking and T-cell redirection. A strategic flow (indicated by arrows) connects tumor debulking to a depicted bone marrow niche, visualizing bridging to HSCT to achieve pre-transplant MRD negativity and post-transplant immune reset. Synergistic targeting integrates small molecule targeted therapies (such as BTK and BCL-2 inhibitors) to modulate the tumor microenvironment, in concert with immune checkpoint inhibition (e.g., PD-1/PD-L1) which reinvigorates exhausted T cells (indicated by a color shift). The overarching sequential network demonstrates how these varied modalities—bridging therapy, transplantation, cytotoxic effectors, checkpoint blockade, and targeted agents—act synergistically to overcome resistance, restore immune function, and achieve a durable response.

To further augment the durability of clinical responses, the concurrent administration of immune checkpoint inhibitors with cellular therapies is being extensively investigated. The upregulation of exhaustion markers, such as PD-1, TIM-3, and LAG-3, on infused CAR-T cells or endogenous T-cells engaged by BsAbs remains a primary mechanism of immune evasion (32). Combinatorial regimens utilizing PD-1 or PD-L1 antagonists aim to prevent host-mediated checkpoint suppression within the tumor microenvironment (33). However, claims regarding the outright ‘reversal’ of terminal T-cell exhaustion via checkpoint blockade must be moderated. Current clinical evidence indicates that this synergy remains strictly investigational and highly context-dependent; while dual blockade can transiently reinvigorate partially exhausted cellular cohorts, its capacity to permanently remodel the closed chromatin architecture of terminally exhausted CAR-T cells remains volatile and clinically inconsistent (34). Additionally, the incorporation of targeted small molecule inhibitors, including Bruton tyrosine kinase inhibitors or BCL-2 inhibitors, has demonstrated preclinical and clinical synergy by modulating the tumor milieu, improving T-cell engraftment, and reducing the incidence of severe cytokine release syndrome without compromising antineoplastic efficacy (35) (**Figure 3**).

Sequential utilization of BsAbs and CAR-T therapies presents another sophisticated avenue for disease management. Bispecific molecules offer an off-the-shelf mechanism for rapid tumor debulking, potentially mitigating the risk of high-grade toxicities associated with subsequent CAR-T infusion (36, 37). Furthermore, in the event of post-CAR-T relapse, BsAbs targeting alternative antigens are deployed to circumvent antigen-loss escape mechanisms. The precise chronological sequencing of these modalities is paramount to prevent overlapping toxicities and profound immune effector exhaustion, ensuring that each intervention primes the host environment for subsequent therapeutic action.

**Figure 4:**
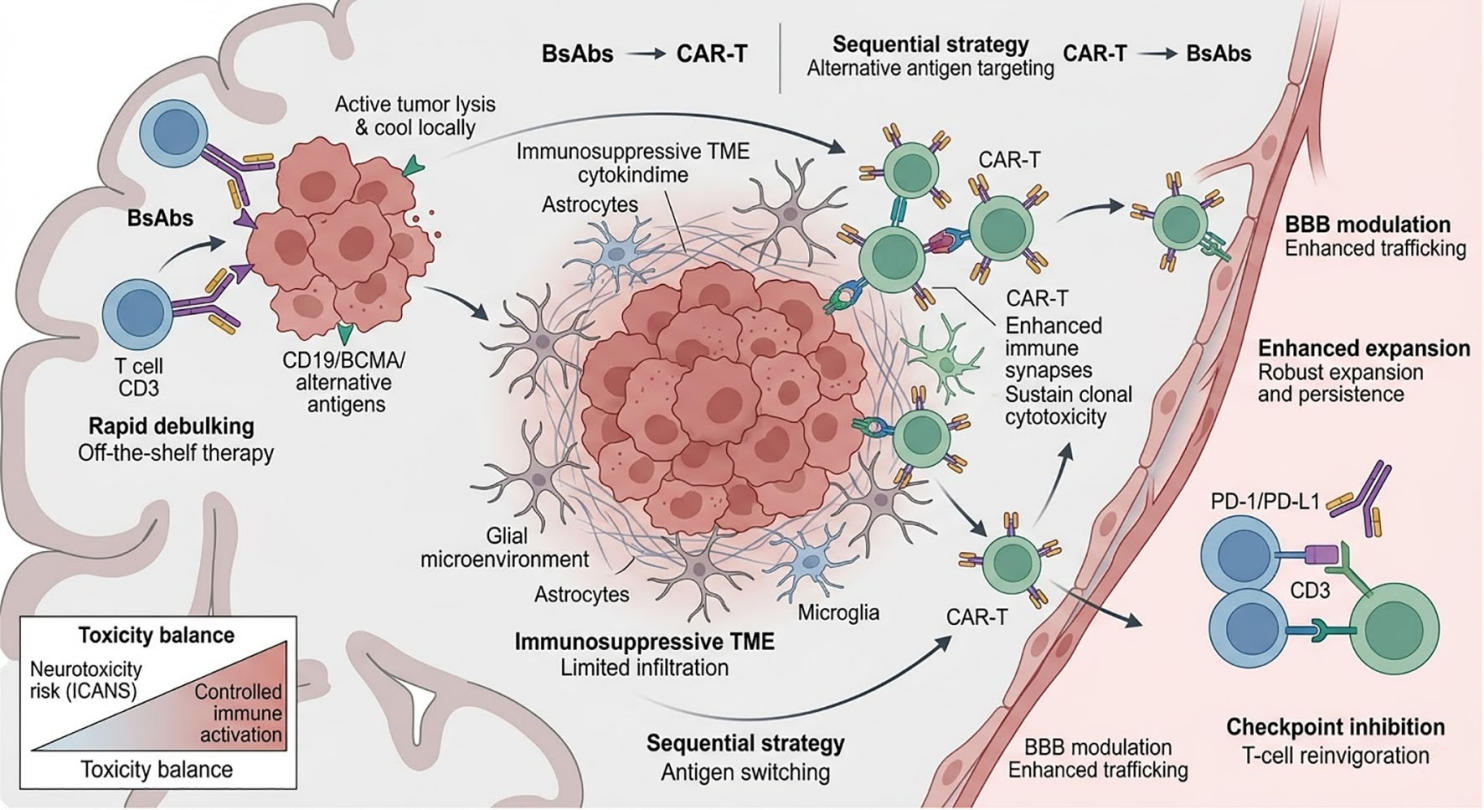
Strategic multimodal engineering to traverse anatomical and immunoregulatory barriers in CNS malignancies. The schematic delineates the rational integration of targeted immunotherapies formulated specifically to bypass the unique neuro-immune axis of intracranial lesions. Left panel (Toxicity Balance): Visualizes the precarious titration between localized therapeutic efficacy and the management of high-grade ICANS. Central panel (Sequential Strategy and Glial Microenvironment): The chronological deployment of BsAbs and multi-specific CAR-T architectures to circumvent antigen-loss escape mechanisms within an immunosuppressive stromal niche composed of reactive astrocytes and microglia. Right panel (Anatomical Penetration and Checkpoint Reinvigoration): Details local delivery mechanisms and engineered BBB transcytosis pathways configured to enhance cellular trafficking, running in tandem with PD-1/PD-L1 checkpoint inhibition to reinvigorate chronically exhausted tumor-infiltrating lymphocytes within the parenchymal core.

These advanced combinatorial frameworks are particularly critical when addressing the profound barriers presented by solid tumors. For instance, in central nervous system malignancies and intricate intracranial lesions, the blood-brain barrier and a profoundly immunosuppressive glial microenvironment severely restrict the infiltration and activation of therapeutic cells (38). By combining targeted immunotherapies with localized delivery mechanisms, transient disruption of the blood-brain barrier, or checkpoint blockade, there is a renewed potential to enhance the trafficking and persistence of engineered T-cells within the brain parenchyma (39–41). This multifaceted approach not only aims to achieve sustained progression-free survival in anatomically sequestered solid tumors but also carefully calibrates the therapeutic window to mitigate the risk of severe immune effector cell-associated neurotoxicity syndrome (**Figure 4**).

## Breaking the wall: immunotherapy in solid tumors

The transition of immunotherapy from hematological malignancies to solid tumors encounters a formidable multidimensional barrier, colloquially termed the tumor microenvironment (42–44). Unlike the intravascular compartment where leukemic cells are readily accessible, solid tumors construct physical, metabolic, and cellular fortresses that actively exclude and suppress infiltrating immune effector cells (45, 46). Overcoming these barriers requires a profound mechanistic understanding of tumor-stroma interactions and the subsequent development of orthogonally engineered T-cells and bispecific molecules (46).

**Figure 5:**
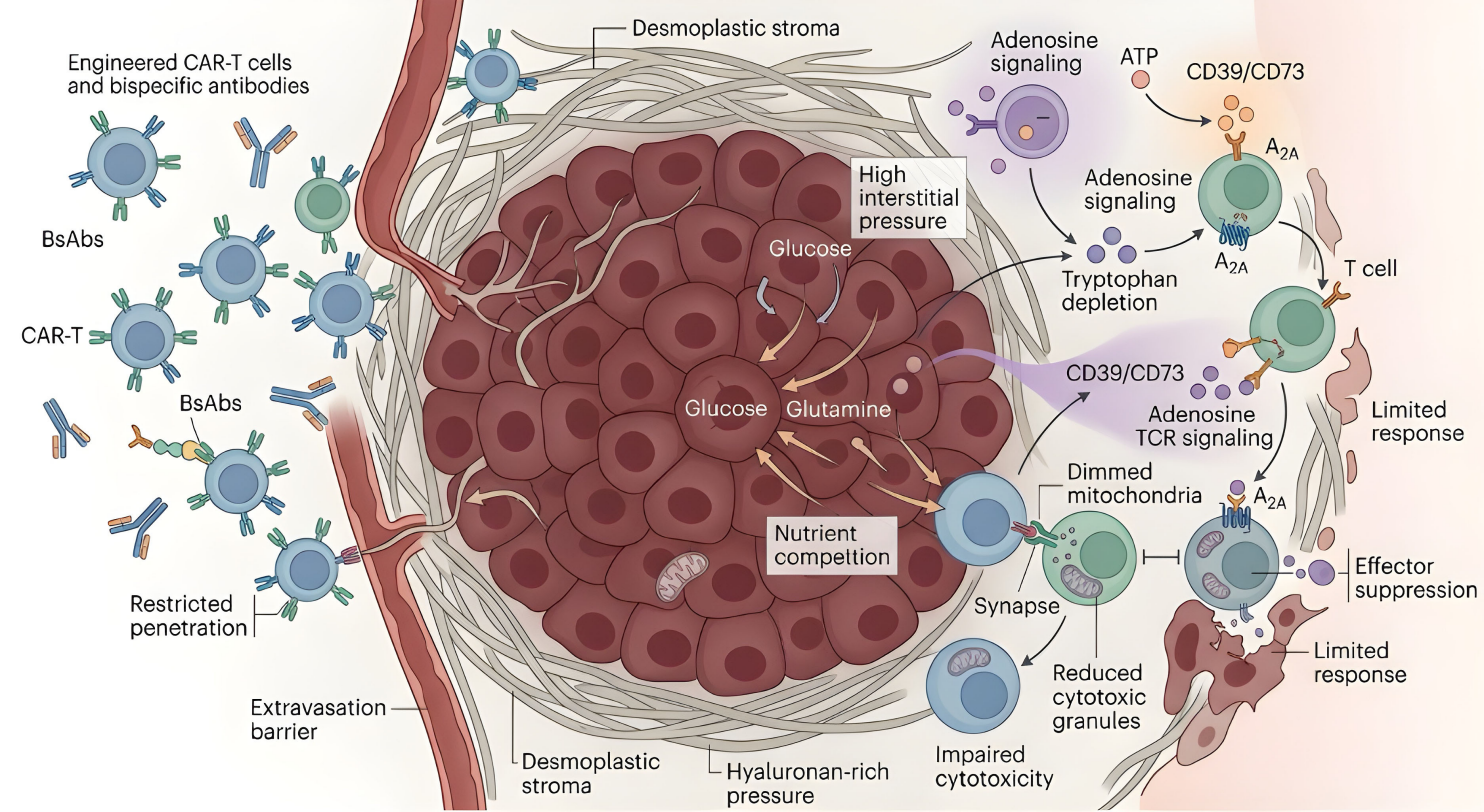
Multilayered physical, metabolic, and immunoregulatory barriers to immunotherapy in the solid tumor microenvironment. Schematic illustrating the integrated obstacles that collectively prevent effective infiltration and activation of CAR-T cells and BsAbs within dense solid tumors. A fibrotic desmoplastic stroma creates a formidable mechanical barrier, acting as an extravasation barrier that restricts penetration of peripheral immune effectors and BsAbs, which is further exacerbated by high interstitial fluid pressure within the tumor core. Moving inward, a zone of intense metabolic competition develops as proliferating tumor cells deplete essential nutrients such as glucose and glutamine, inducing T-cell anergy characterized by dimmed mitochondrial function. T cells that do infiltrate are subjected to a complex network of immunosuppressive metabolites, including kynurenine generated via indoleamine 2,3-dioxygenase (IDO)-mediated tryptophan depletion, and adenosine signaling—produced through the sequential conversion of ATP—which potently suppresses T-cell receptor (TCR) signaling by binding to A2A receptors. These cumulative barriers lead to widespread effector suppression and impaired cytotoxicity, ultimately resulting in only a limited therapeutic response at the tumor margins and necessitating advanced engineering to overcome tumor microenvironment resistance. ATP, adenosine triphosphate; BBB, blood-brain barrier; CNS, central nervous system; dnTGFβ-RII, dominant-negative transforming growth factor-beta receptor type II; IDO, indoleamine 2,3-dioxygenase; MDSCs, myeloid-derived suppressor cells; TAMs, tumor-associated macrophages; TCR, T-cell receptor; TME, tumor microenvironment; Tregs, regulatory T-cells.

A primary physical impediment is the dense desmoplastic stroma and aberrant extracellular matrix characteristic of many solid tumors (47). Pathological cross-linking of collagen and the overproduction of hyaluronan generate elevated interstitial fluid pressure, which severely restricts the extravasation and parenchymal penetration of macromolecular BsAbs and CAR-T cells (48). Concurrently, the metabolic landscape of the tumor microenvironment is inherently hostile. Rapidly proliferating malignant cells outcompete infiltrating lymphocytes for essential nutrients such as glucose and glutamine, inducing an anergic state in T-cells. This metabolic starvation is exacerbated by the accumulation of immunosuppressive metabolites (49). For instance, the upregulation of indoleamine 2,3-dioxygenase catalyzes the depletion of local tryptophan, while the CD39/CD73 ectonucleotidase axis converts extracellular ATP into adenosine, which binds to A2A receptors on T-cells to potently extinguish T-cell receptor signaling and impair cytolytic degranulation (**Figure 5**).

Beyond physical and metabolic restrictions, solid tumors recruit an array of immunosuppressive cellular subsets, predominantly regulatory T-cells, myeloid-derived suppressor cells, and M2-polarized tumor-associated macrophages (50–52). These cellular mediators sustain immune tolerance through the constitutive secretion of suppressive cytokines, most notably transforming growth factor-beta and IL-10 (53). Transforming growth factor-beta signaling directly antagonizes cytotoxic T-lymphocyte proliferation and downregulates the expression of essential cytolytic molecules, such as perforin and granzymes, via the SMAD intracellular pathway (54, 55). To counteract these potent inhibitory signals, next-generation cellular therapies are being armed with dominant-negative receptors. By engineering CAR-T cells to co-express a dominant-negative transforming growth factor-beta receptor type II, the immunosuppressive signaling cascade is uncoupled, allowing the engineered T-cells to maintain robust effector functions even within a highly hostile cytokine milieu.

Antigen heterogeneity and lineage plasticity present another profound challenge in solid oncology (56). In central nervous system malignancies such as glioblastoma, the heterogeneous expression of tumor-associated antigens like EGFRvIII (Epidermal Growth Factor Receptor variant III), IL-13R alpha 2, and HER2 (Human Epidermal Growth Factor Receptor 2) frequently leads to rapid antigen escape following single-target directed therapy (57–60). Crucially, high-profile clinical trials evaluating first-generation EGFRvIII-directed CAR-T cells in glioblastoma have historically yielded negative results regarding long-term clinical efficacy. While transient target alignment and localized inflammatory responses were achieved, these trials ultimately failed to significantly prolong overall survival. The underlying causes of these negative outcomes are rooted in rapid, multi-clonal antigen escape and the active down-regulation of the EGFRvIII variant post-infusion, allowing pre-existing antigen-negative tumor clones to aggressively drive disease progression. This clinical reality clearly demonstrates that a solitary antigenic focus is insufficient to breach the dense spatial and cellular heterogeneity of the glioblastoma microenvironment (60, 61). Concurrently, the engineering of multivalent or tandem CAR constructs, alongside logic-gated synthetic circuits incorporating AND/OR Boolean logic, aims to increase tumor specificity while mitigating on-target, off-tumor toxicities against healthy tissues.

**Figure 6:**
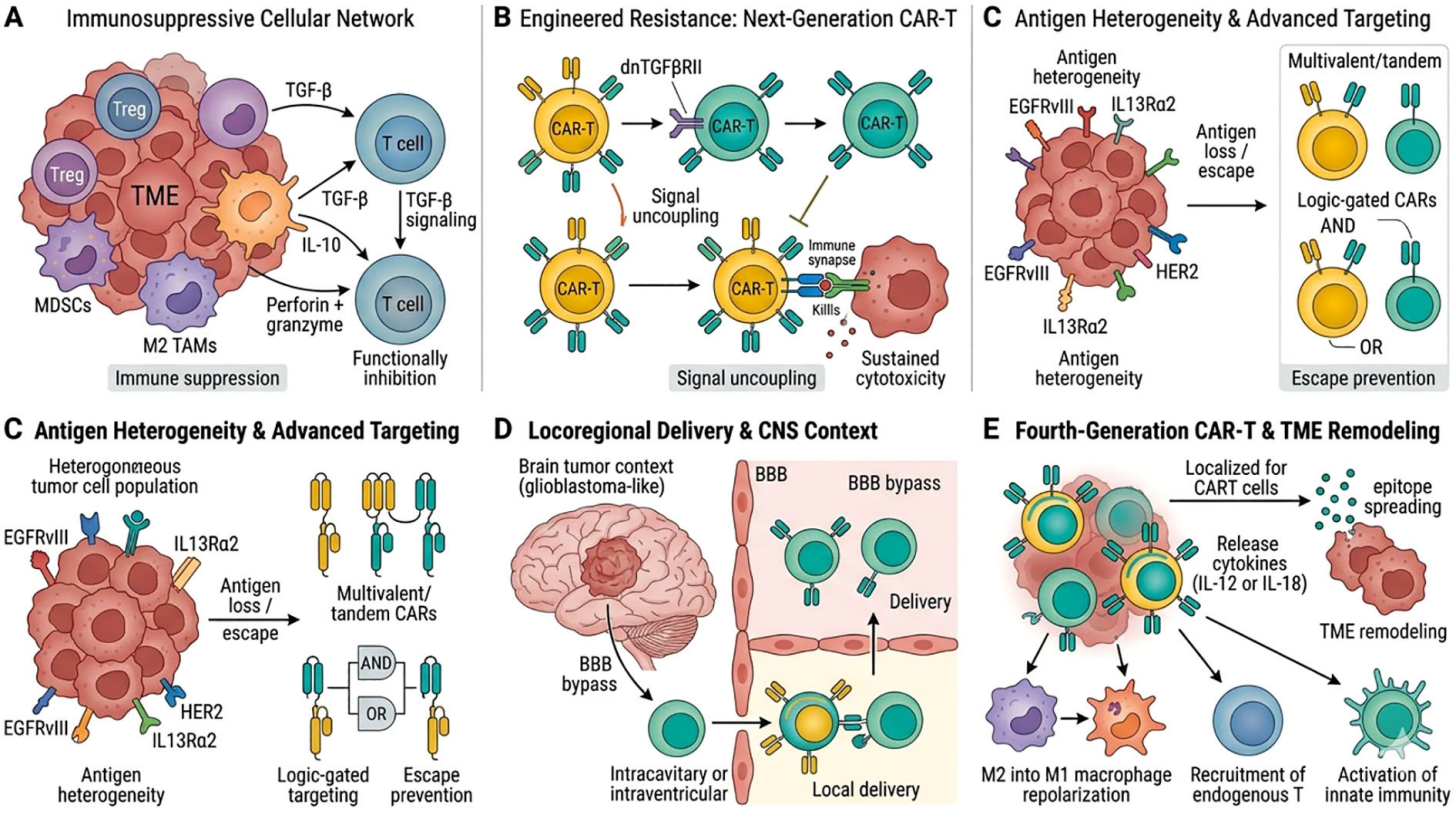
Multilayered solid tumor immunosuppression and next-generation engineered CAR-T strategies to overcome therapeutic resistance. **(a)** Highly complex and immunosuppressive cellular networks dominate the solid tumor microenvironment (TME). Regulatory T cells (Tregs), myeloid-derived suppressor cells (MDSCs), and M2 tumor-associated macrophages (M2 TAMs) constitute a suppressive milieu. These cells secrete potent immune inhibitory cytokines, including transforming growth factor-beta (TGF-β) and IL-10. The engagement of TGF-β with its receptor on a neighboring effector T cell activates the SMAD signaling pathway, leading to powerful effector molecule downregulation, including perforin and granzyme, resulting in functional suppression. **(b)** An engineering strategy to introduction resistance to suppressive cytokines. Next-generation CAR-T cells are genetically engineered to express a dominant-negative TGF-β receptor (dnTGFβRII). This mutation uncouples inhibitory signaling, preventing effector molecule downregulation. These modified dnTGFβRII CAR-T cells sustain robust cytotoxic activity, forming functional immune synapses and actively killing tumor cells. **(c)** Advanced targeting strategies to address antigen heterogeneity and prevent antigen loss or escape in heterogeneous tumor cell populations. Two complementary approaches are visualized. One presentation focuses on logic-gated targeting utilizing Boolean AND/OR logic circuits to provide precise control over activation and reduce off-tumor toxicity. A complementary strategy employs multivalent or tandem CAR architectures to target multiple distinct antigens, such as EGFRvIII, IL13Rα2, and HER2, enabling broader coverage and minimizing escape of antigen-negative variants. **(d)** The unique central nervous system (CNS) context, visualized with a glioblastoma-like brain tumor and blood-brain barrier (BBB). CAR-T cells are delivered directly via intracavitary or intraventricular routes (“local delivery”) to bypass systemic BBB limitations. **(e)** Fourth-generation armored CAR-T cells localized for inducible release of cytokines such as IL-12 or IL-18. Localized cytokine release remodels the TME, initiating a cascade of effects: M2 to M1 macrophage repolarization, recruitment of endogenous T cells, and activation of innate immunity. This drives an “epitope spreading” effect for broad and durable anti-tumor immunity. Minimal labels and professional biomedical colors are used throughout. The ultra-high resolution vector image contains no title or watermarks.

The transition of immunotherapy from hematological malignancies to solid tumors encounters a formidable multidimensional barrier, colloquially termed the tumor microenvironment. To outline these obstacles systematically, they can be categorized into three distinct layers: physical extracellular structures, metabolic competitive pathways, and cellular immunosuppressive networks. Additionally, fourth-generation armored CAR-T cells are engineered to secrete transgenic cytokines, such as IL-12 or IL-18, in a strictly antigen-inducible manner to promote local epitope spreading (62, 63). While this approach represents an active investigational advancement, its clinical maturity remains in its infancy; clinical trial data evaluating armored cellular products in solid malignancies indicate notable volatility in balancing local anti-tumor efficacy against systemic leaky cytokine toxicities, meaning its safety margins require extensive validation before it can be integrated into standardized regimens (64, 65) (**Figure 6**).

## Next-generation molecular innovations

The relentless pressure of immune evasion within the solid tumor microenvironment has catalyzed a paradigm shift from conventional CAR designs toward highly sophisticated, synthetically engineered immune circuits (66). Next-generation molecular innovations are fundamentally driven by the need to dynamically sense, adapt to, and therapeutically remodel the immunosuppressive niche while maintaining stringent spatial and temporal control over immune effector activation.

To circumvent the pervasive issue of antigen heterogeneity, which is profoundly observed in high-grade central nervous system malignancies such as glioblastoma, researchers are developing multi-antigen targeting strategies empowered by Boolean logic gating (67). Traditional single-target CARs frequently succumb to target down-regulation and antigen escape. In contrast, “AND-gate” synthetic circuits, such as those utilizing the synthetic Notch (synNotch) receptor system, require dual-antigen recognition for full T-cell activation (68). Mechanistically, the engagement of the first, ubiquitously expressed local antigen by the synNotch receptor induces an intramembrane proteolytic cleavage of its intracellular domain. This cleaved transcription factors (TFs) subsequently translocates to the nucleus to drive the localized expression of a secondary, canonical CAR directed against a more specific, heterogeneously expressed tumor-associated antigen. This sequential activation mechanism ensures that potent cytolytic function is exclusively unleashed in the presence of both antigens, drastically widening the therapeutic window and mitigating on-target, off-tumor toxicity against healthy neural parenchyma. Despite this elegant mechanistic design, synNotch circuits present significant, unresolved safety concerns that complicate their clinical translation (68, 69). The continuous, irreversible genomic integration of synthetic, non-human TFs introduces an inherent risk of insertional mutagenesis and genomic instability. Furthermore, the constitutive expression of these foreign synthetic TFs can trigger host-mediated immunogenicity, leading to the rapid clearance of the therapeutic cells (70). Crucially, when using conventional retroviral or lentiviral delivery vectors, the risk of clonal dominance and potential oncogenic transformation remains a critical barrier that must be rigorously addressed before these synthetic biology platforms can be safely introduced into human trials (71).

Furthermore, the structural architecture of the CAR itself is undergoing significant intracellular optimization. In the context of this review, we utilize the term ‘fifth-generation CAR’ specifically to describe constructs that incorporate an additional truncated intracellular domain derived from cytokine receptors, most notably the IL-2 receptor beta chain, though it is critical to acknowledge that terminology regarding CAR generations is still evolving and lacks universal consensus in the field (72). While some groups reserve this designation for multi-antigen targeting architectures combined with intrinsic safety switches, the integration of the IL-2 receptor beta domain represents a distinct molecular strategy to overcome metabolic exhaustion. While conventional second-generation CARs rely on CD28 or 4-1BB costimulation in tandem with the CD3ζ chain to activate the PI3K/AKT and TRAF signaling cascades, fifth-generation constructs incorporate an additional truncated intracellular domain derived from cytokine receptors, most notably the IL-2 receptor beta chain (73). Upon antigen recognition, this integrated domain orchestrates the localized recruitment and activation of Janus kinases (JAKs), which subsequently phosphorylate STAT (Signal Transducer and Activator of Transcription) 3 and STAT5 TFs (74, 75). Traditional single-target CARs frequently succumb to target down-regulation and antigen escape. In contrast, ‘AND-gate’ synthetic circuits, such as those utilizing the synNotch receptor system, require dual-antigen recognition for full T-cell activation (76). Functionally, this sequential processing ensures that potent cytolytic activation is restricted strictly to tissue boundaries displaying both antigens, thereby expanding the therapeutic window and preventing on-target, off-tumor destruction of healthy parenchyma. Upon antigen recognition, this integrated domain orchestrates the localized recruitment and activation of JAKs, which subsequently phosphorylate STAT3 and STAT5 TFs (77). This engineered convergence is designed to optimize the transcriptional landscape of the cellular product. However, as these fifth-generation constructs are currently evaluated within preclinical frameworks or early Phase I safety studies, claims regarding their capacity to expand entirely independent of systemic cytokine support remain investigational, and long-term cellular exhaustion patterns under chronic antigen challenge require extensive verification.

In the realm of BsAbs, molecular engineering is heavily focused on achieving conditional activation and traversing anatomical barriers. A critical mechanistic innovation is the development of protease-activatable or “masked” bispecific T-cell engagers (78). Structurally, the antigen-binding domains of these molecules are sterically occluded by an inhibitory peptide mask linked via a specifically designed protease-cleavable linker. The T-cell engaging CD3-binding domain remains functionally inert in the systemic circulation, thereby preventing systemic cytokine release syndrome and off-target T-cell margination. Upon permeating the tumor microenvironment, highly localized tumor-associated proteases, such as specific matrix metalloproteinases or urokinase-type plasminogen activators, cleave the linker (79). This site-specific proteolytic unmasking restores the binding affinity of the bispecific molecule, triggering localized cross-linking of T-cells exclusively to malignant cells.

**Figure 7:**
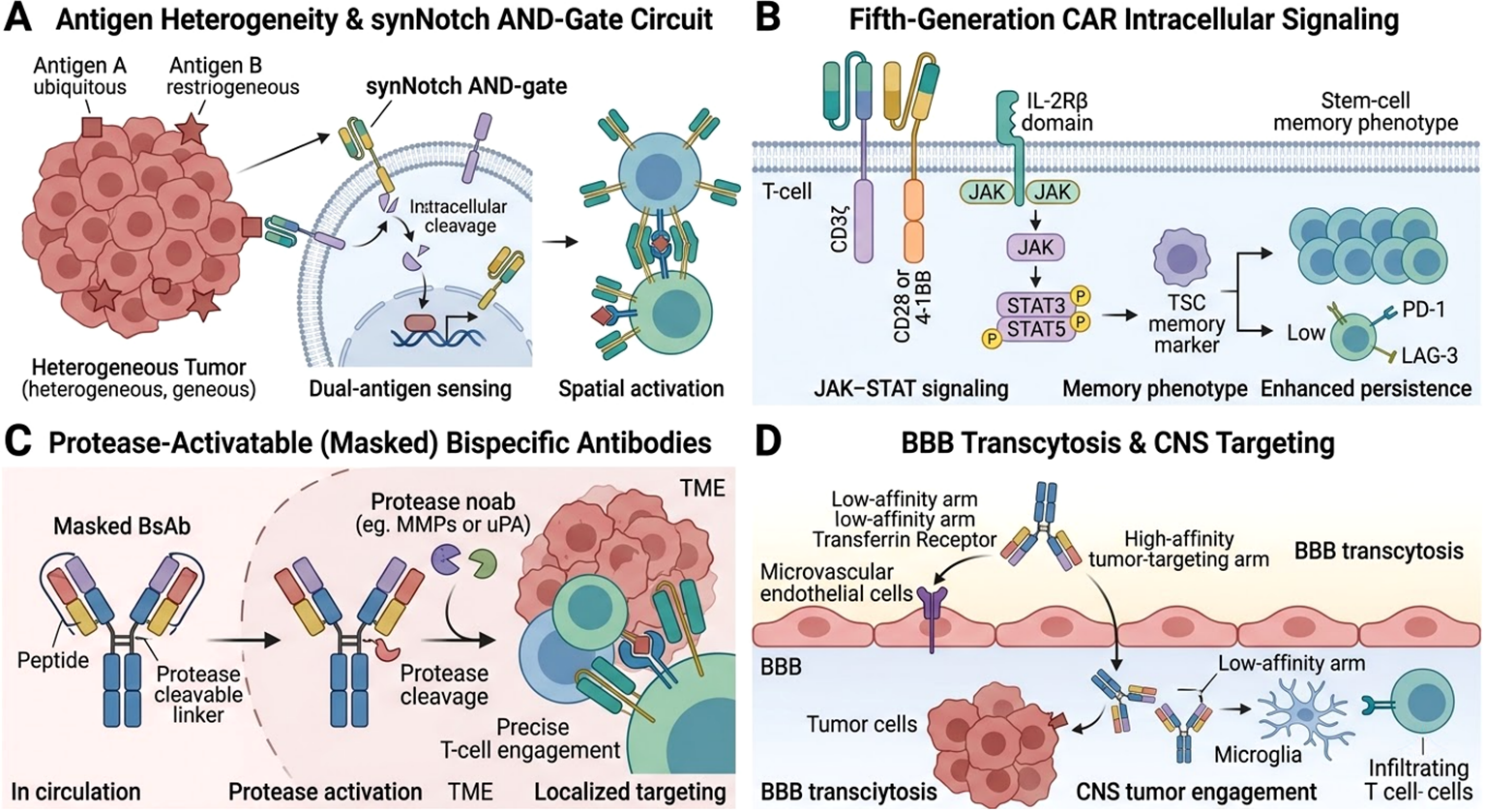
Next-generation synthetic immune circuits for cancer therapy. **(A)** synNotch AND-gate circuit to overcome antigen heterogeneity. Ubiquitous Antigen A triggers synNotch cleavage, releasing a TF that customizes expression of a CAR against heterogeneous Antigen **(B)** This dual-antigen sensing ensures spatial activation and prevents antigen escape. **(B)** customized intracellular domain for fifth-generation CAR T-cell signaling. A customizable IL-2Rβ domain recruits JAK, initiating JAK-STAT signaling to rewire memory pathways toward a stem-cell memory phenotype (TSC memory marker) and enhance persistence by maintaining low expression of exhaustion markers PD-1 and LAG-3. **(C)** masked BsAbs. Bispecific antibody (BsAb) binding arms are covered by a peptide and connected by a protease-cleavable linker. Cleavage by specific proteases (e.g., MMPs or uPA) in the tumor microenvironment (TME) removes the mask, activating the molecule for localized targeting and precise T-cell engagement. **(D)** Blood-Brain Barrier (BBB) transcytosis strategy for CNS targeting. An engineered bispecific antibody with a low-affinity arm binding the Transferrin Receptor undergoes receptor-mediated transcytosis across brain microvascular endothelial cells forming the BBB. This allows targeted delivery into the brain parenchyma for subsequent high-affinity CNS tumor engagement and microglia recruitment, overcoming the anatomically restricted environment. Vector illustrations at ≥6000 px width without watermarks. CRS, cytokine release syndrome; GM-CSF, granulocyte-macrophage colony-stimulating factor; ICANS, immune effector cell-associated neurotoxicity syndrome; IL, interleukin; JAK/STAT, Janus kinase/signal transducer and activator of transcription; MMPs, matrix metalloproteinases; synNotch, synthetic Notch; TF, transcription factor; TfR, transferrin receptor; TNF-$\alpha$, tumor necrosis factor-alpha; uPA, urokinase-type plasminogen activator.

Concurrently, the architectural design of these multispecific molecules is being optimized to overcome the blood-brain barrier for the treatment of primary intracranial tumors and metastatic lesions. By engineering asymmetric immunoglobulin formats where one fragment antigen-binding arm possesses a finely tuned, low-affinity domain for a transcytosis-mediating receptor—such as the transferrin receptor—these molecules can exploit receptor-mediated transcytosis across the brain microvascular endothelial cells (80). Once transcytosed into the brain parenchyma, the contralateral high-affinity arm engages the tumor-specific antigen, redirecting both resident microglia and infiltrating systemic lymphocytes against the malignant focus (81). This precise molecular engineering ensures that the potent immunological synapse is formed strictly within the confined tumor bed, representing a sophisticated mechanistic approach to managing anatomically sequestered and molecularly complex neoplasms (**Figure 7**).

Once transcytosis into the brain parenchyma, the contralateral high-affinity arm engages the tumor-specific antigen, redirecting both resident microglia and infiltrating systemic lymphocytes against the malignant focus (82). This precise molecular engineering ensures that the potent immunological synapse is formed strictly within the confined tumor bed, representing a sophisticated mechanistic approach to managing anatomically sequestered and molecularly complex neoplasms. However, it is vital to balance these elegant synthetic frameworks against current translational reality: the capacity of these next-generation BsAbs to efficiently ‘traverse the BBB’ via transferrin receptor (TfR) transcytosis is almost entirely based on early mouse data, and its clinical feasibility and safety in humans remain unproven (83). Translating this approach into routine clinical practice faces major translational barriers, including a systemic ‘sink effect’ driven by high TfR expression on peripheral hematopoietic lineages, potential on-target cerebrovascular endothelial toxicities, and strict cross-species affinity discrepancies that obscure predictable human safety profiles. Therefore, these approaches must be carefully categorized as visionary preclinical strategies requiring rigorous clinical verification.

## Clinical outcomes and toxicity management

The unprecedented clinical outcomes achieved by CAR-T cells and BsAbs, frequently measured by profound improvements in overall survival and progression-free survival, are intrinsically coupled to exceptionally narrow therapeutic indices. The primary dose-limiting toxicity, cytokine release syndrome, is fundamentally driven by a hyper-inflammatory positive feedback loop rather than the direct cytolytic action of the engineered cells (84, 85). Upon target antigen engagement, engineered T-cells secrete primary cytokines, predominantly interferon-gamma and tumor necrosis factor-alpha (84). These molecules serve as potent activators of the bystander innate immune system, specifically targeting reticulo-endothelial macrophages and circulating monocytes. This interaction triggers a massive secondary efflux of pivotal pyrogenic cytokines, most notably IL-6 and IL-1 beta (86). The systemic dissemination of IL-6 allows it to bind to both membrane-bound and soluble IL-6 receptors, initiating widespread endothelial cell activation via the trans-signaling pathway. This intracellular cascade culminates in profound vascular hyperpermeability, capillary leak syndrome, and consumptive coagulopathy, which manifest clinically as refractory hypotension, severe hypoxia, and ultimately multi-organ dysfunction.

**Figure 8:**
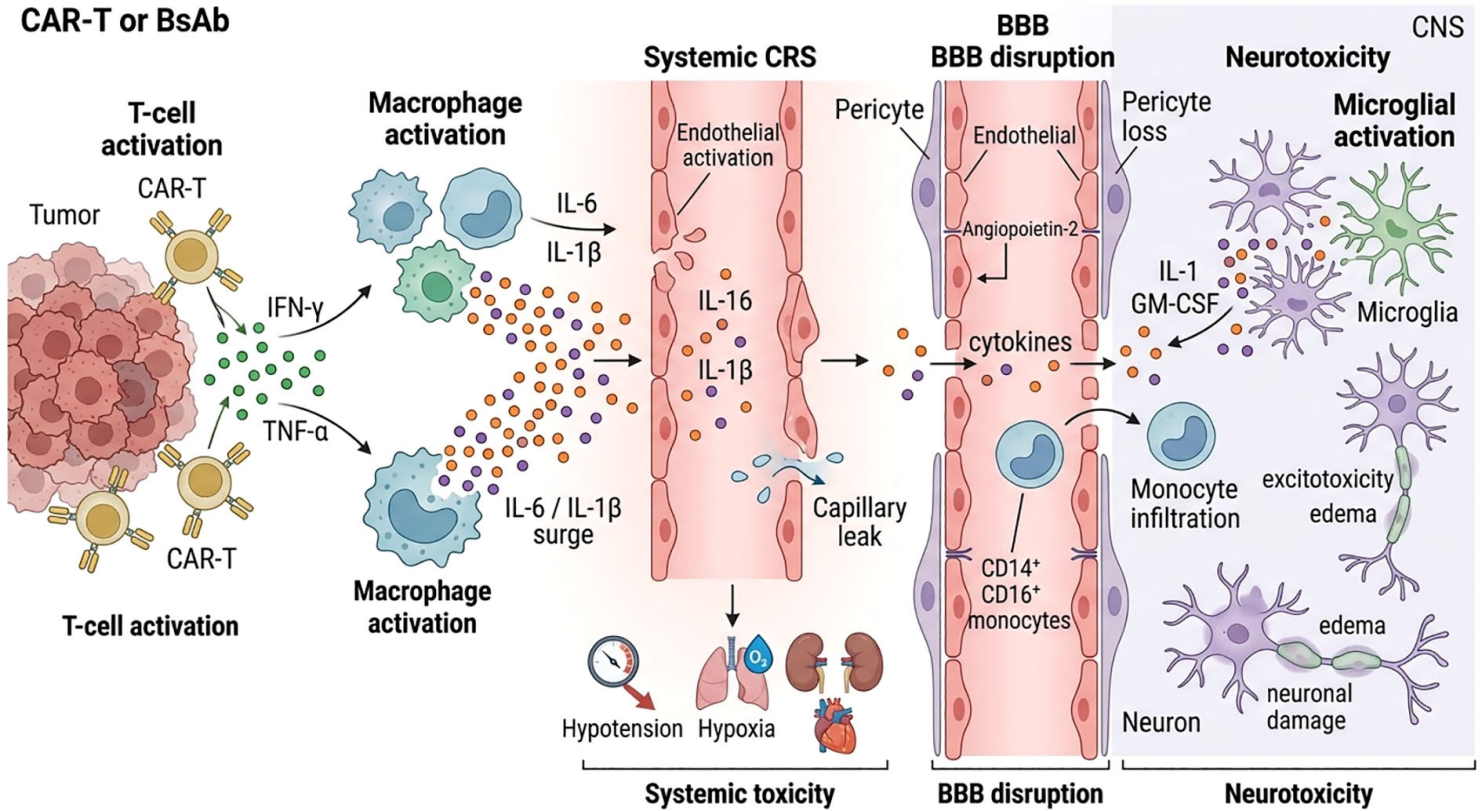
Pathophysiological mechanisms from T-cell therapy to neurotoxicity. CAR-T or Bispecific Antibody (BsAb) engage tumor cells, initiating T-cell activation, which leads to the release of IFN-γ and TNF-α. This initiates a positive feedback loop of innate immune amplification by activating macrophages and monocytes, which secrete massive surges of pro-inflammatory cytokines, predominantly IL-6 and IL-1β. These high cytokine levels enter the systemic circulation, driving acute endothelial cell activation in capillary endothelial cells, which manifest in CRS-related Systemic Toxicity, indicated by Capillary Leak, Hypotension, Hypoxia, and Organ Dysfunction. In the final phase of this cascade, Systemic CRS disruption compromises the blood-brain barrier (BBB), characterized by angiopoietin-2 upregulation, and loss of the protective pericyte layer. Compromise facilitates the neurovascular transit of CD14+ CD16+ inflammatory monocytes and cytokines, including IL-6 and IL-1β, from the circulation into the CNS compartment. This triggers microglial activation to secrete localized cytokines such as IL-1 and GM-CSF, propagating profound microglial activation and subsequent excitotoxic neuronal damage, edema, and clinical ICANS Neurotoxicity. High-resolution vector art with sharp rectangular edges and balanced margins.

Concurrent with or sequentially following systemic inflammation, immune effector cell-associated neurotoxicity syndrome presents a profound and life-threatening clinical challenge. The pathophysiological mechanism of this neurotoxicity is intimately linked to the structural and functional disruption of the blood-brain barrier. Systemic endothelial activation leads to the marked upregulation of angiopoietin-2 and the concomitant loss of protective pericyte architecture within the central nervous system microvasculature (87). This structural vulnerability facilitates the pathological transit of hyperactivated CD14+ CD16+ inflammatory monocytes and high-molecular-weight circulating cytokines directly into the cerebrospinal fluid (88). Crucially, while IL-6 is the primary driver of systemic toxicity, elevated levels of IL-1 and GM-CSF within the central nervous system compartment are strongly implicated as key molecular correlators of ICANS, though their precise mechanistic hierarchy and direct causality in human clinical cohorts remain under active investigation (89–91). These specific cytokines propagate profound microglial activation and induce excitotoxic neural damage, manifesting clinically as expressive aphasia, rapidly fluctuating encephalopathy, and potentially fatal cerebral edema. The pharmacological management of these distinct toxicities has evolved from empiric, broad-spectrum immunosuppression to highly targeted mechanistic interventions. To counteract this phenomenon, agents such as anakinra, a recombinant IL-1 receptor antagonist, are increasingly utilized. Anakinra possesses the capacity to penetrate the central nervous system and competitively inhibit IL-1 signaling at the level of resident glia (92). However, its systemic efficacy and capacity to fully reverse severe, refractory neurotoxicity represent an emerging clinical paradigm that is not yet universally standardized or validated in large-scale randomized controlled trials. These specific cytokines propagate profound microglial activation and induce excitotoxic neural damage, manifesting clinically as expressive aphasia, rapidly fluctuating encephalopathy, and potentially fatal cerebral edema (**Figure 8**).

The pharmacological management of these distinct toxicities has evolved from empiric, broad-spectrum immunosuppression to highly targeted mechanistic interventions (93). While high-dose systemic corticosteroids remain a backbone for mitigating unchecked immune hyperactivation, their indiscriminate use risks the induction of T-cell apoptosis and the abrogation of long-term anti-tumor efficacy (94). Consequently, targeted monoclonal antibodies such as tocilizumab, a competitive inhibitor of the IL-6 receptor, have been established as the standard of care for rapidly reversing systemic capillary leak (95). However, the macromolecular structure of tocilizumab precludes its substantial penetrance across the blood-brain barrier (96). Crucially, while IL-6 is heavily involved in systemic toxicity cascades, elevated profiles of IL-1 and GM-CSF within the central nervous system compartment are strongly associated with the development of ICANS, though the precise chronological hierarchy and causal drivers of human neurotoxicity remain partially understood and are the subject of evolving evidence (97, 98). Consequently, targeted monoclonal antibodies such as tocilizumab, a competitive inhibitor of the IL-6 receptor, have been established as the standard of care for rapidly reversing systemic capillary leak (99). While peripheral IL-6 receptor blockade by tocilizumab can theoretically result in a transient accumulation of unbound, free serum IL-6 that exhibits limited penetrance across an intact blood-brain barrier, current clinical management guidelines indicate that its administration does not consistently correlate with an automatic, direct exacerbation of neurotoxicity, though careful neurological monitoring during the systemic resolution phase remains mandatory (100). To counteract this phenomenon, agents such as anakinra, a recombinant IL-1 receptor antagonist, are increasingly utilized. Anakinra possesses the capacity to penetrate the central nervous system and competitively inhibit IL-1 signaling at the level of resident glia (101).

To achieve preemptive toxicity management without relying solely on rescue medications, synthetic immunology has successfully integrated pharmacological control mechanisms directly into the cellular architecture. Small molecule tyrosine kinase inhibitors, most notably dasatinib, have been ingeniously repurposed as reversible pharmacologic off-switches. Mechanistically, dasatinib rapidly and potently inhibits the lymphocyte-specific protein tyrosine kinase, an enzyme strictly required for the initial phosphorylation of the CD3-zeta immunoreceptor tyrosine-based activation motifs immediately following antigen ligation (102). To achieve preemptive toxicity management without relying solely on rescue medications, synthetic immunology has successfully integrated pharmacological control mechanisms directly into the cellular architecture. Small molecule tyrosine kinase inhibitors, most notably dasatinib, have been ingeniously repurposed as reversible pharmacologic off-switches (103, 104). Mechanistically, dasatinib rapidly and potently inhibits the lymphocyte-specific protein tyrosine kinase (105). However, presenting dasatinib as a universally reliable or standard-of-care ‘pharmacologic off-switch’ overstates its current clinical maturity, as this approach remains predominantly in the preclinical stage and limited to isolated clinical case reports. In routine clinical practice, the deployment of such small-molecule tyrosine kinase inhibitors is fraught with significant translational caveats (103, 106). These include the precarious risk of tumor lysis syndrome upon abrupt CAR-T reactivation after drug withdrawal, a severe ‘rebound’ cytokine release syndrome driven by the sudden release of accumulated intracellular cytokine reservoirs, and evidence of incomplete or transient inhibition of certain hyper-stimulated, high-affinity CAR-T cell sub-populations. Therefore, while conceptually elegant, its routine translational application requires extreme caution (107).

## Future directions and challenges

The trajectory of precision immuno-oncology is increasingly defined by the transition from reactive toxicity management and empirical combinatorial trials toward predictive biomarker discovery and advanced bioengineering. A primary future challenge lies in deciphering the highly complex spatial and temporal dynamics of the tumor microenvironment to predict primary and acquired resistance. Current predictive models relying on singular biomarkers, such as programmed death-ligand 1 expression or tumor mutational burden, frequently fail to capture the multidimensional mechanisms of immune evasion. Future directions heavily emphasize the integration of single-cell RNA sequencing and high-plex spatial transcriptomics to delineate the exact topographical distribution of immune effectors and immunosuppressive myeloid compartments within the tumor stroma. Mechanistically, researchers are focusing on the epigenetic landscape of T-cell exhaustion, specifically the irreversible chromatin remodeling driven by TFs such as TOX and the NR4A family (108, 109). Identifying epigenetic signatures of terminal exhaustion in pre-infusion apheresis products or post-infusion circulating lymphocytes will allow for the prospective identification of patients destined to fail autologous cellular therapy, prompting the early integration of epigenetic modifiers, such as hypomethylating agents, to rescue T-cell plasticity (109, 110).

Simultaneously, the prohibitive cost, manufacturing bottlenecks, and frequent out-of-specification failures associated with autologous CAR-T therapys necessitate a paradigm shift toward allogeneic, “off-the-shelf” platforms (111–113). However, the deployment of healthy donor-derived T-cells introduces profound immunological barriers, specifically graft-versus-host disease and host-versus-graft rejection. Overcoming these barriers relies on multiplexed genomic engineering, predominantly utilizing CRISPR-Cas9 ribonucleoprotein complexes (114, 115). To mitigate graft-versus-host disease, the endogenous T-cell receptor alpha constant locus is disrupted, permanently abrogating the expression of the alloreactive alpha-beta T-cell receptor. Concurrently, to evade host-mediated allo-rejection, the beta-2 microglobulin gene is knocked out, eliminating the surface expression of human leukocyte antigen class I molecules and hiding the engineered cells from the recipient’s cytotoxic CD8-positive T-cells (116, 117). Because the absence of human leukocyte antigen class I paradoxically triggers profound natural killer cell-mediated lysis via “missing-self” recognition mechanisms, advanced allogeneic constructs are now being engineered to constitutionally express non-polymorphic human leukocyte antigen-E or human leukocyte antigen-G, which bind to inhibitory receptors on host natural killer cells, thereby achieving a state of localized immunoprivilege.

Beyond *ex vivo* cellular engineering, the ultimate conceptual leap in the field involves the *in vivo* generation of CAR-T therapys, bypassing the need for complex *ex vivo* lymphodepletion, cell expansion, and reinfusion (118–121). Beyond *ex vivo* cellular engineering, the ultimate conceptual leap in the field involves the *in vivo* generation of CAR-T therapys, bypassing the need for complex ex vivo lymphodepletion, cell expansion, and reinfusion (122). However, it is critical to contextualize that these *in vivo* programming strategies reside strictly within the early preclinical bench stage, and presenting them as near-term clinical realities overstates their current translational maturity (6). While these concepts are intellectually exciting, migrating from early proof-of-concept murine studies to human clinical testing faces formidable and unmitigated biological barriers. Mechanistically, lipid nanoparticles are conjugated with targeting ligands, such as anti-CD8 monoclonal antibodies, and formulated to encapsulate nucleoside-modified messenger RNA encoding the CAR (123). Although this transient *in vivo* approach is designed to circumvent the replicative senescence inherently induced by prolonged *ex vivo* culture, current preclinical iterations are severely constrained by low targeted delivery efficiency into primary secondary lymphoid organs, transient and unpredictable transgene expression persistence, systemic immunogenicity directed against the liposomal or viral delivery components, and a precarious risk of genotoxicity or insertional mutagenesis if off-target viral integration occurs within non-T-cell lineages or stem cell compartments (124). Alternatively, targeted lentiviral or adeno-associated viral vectors are being investigated for stable genomic integration *in vivo*.

**Figure 9:**
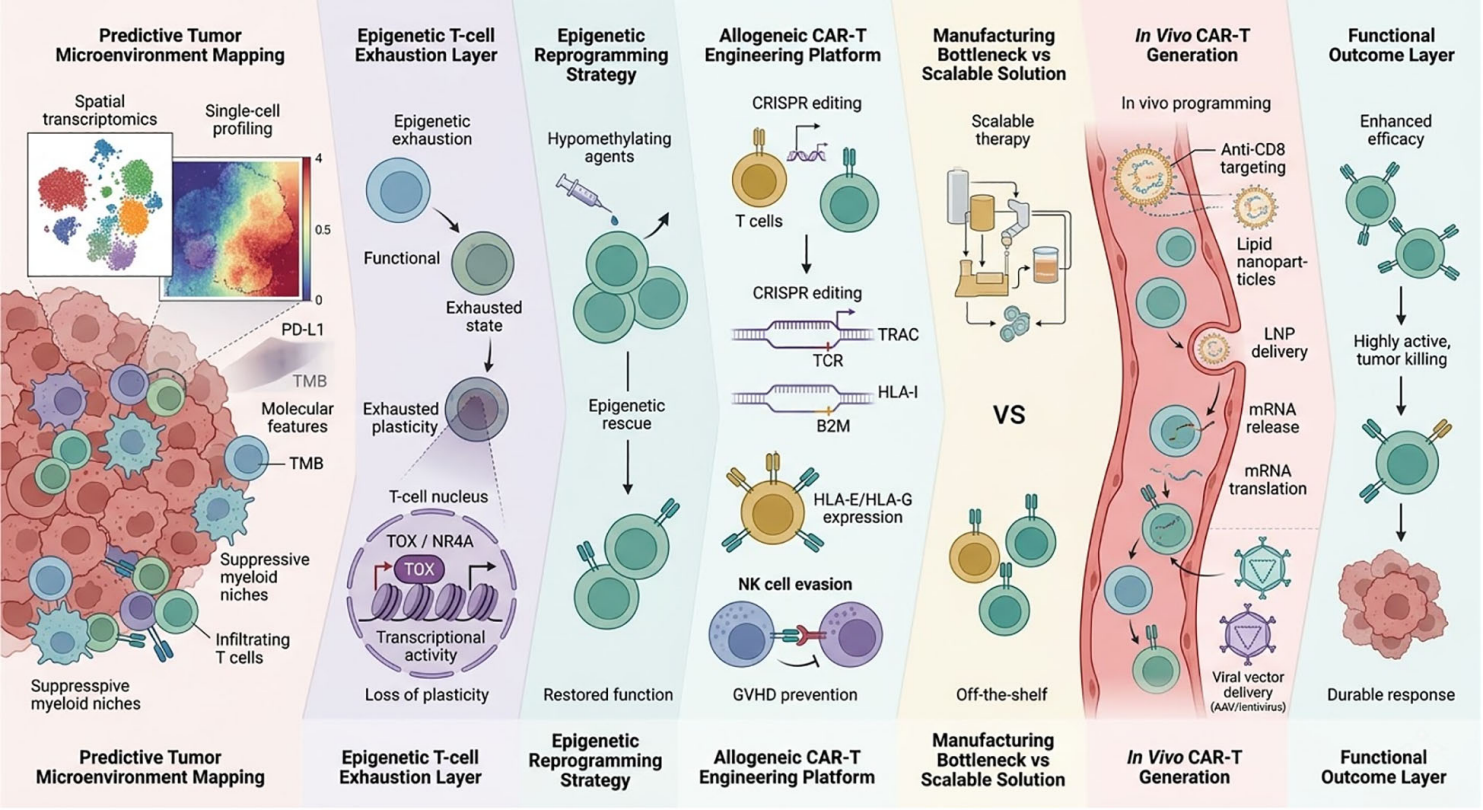
Predictive, engineered, and scalable next-generation immuno-oncology. Integrated schematic illustrating the integrated landscape of future immuno-oncology, shifting from conventional reactive treatments toward predictive, highly engineered, and scalable immunotherapy ecosystems. On the far left, predictive tumor microenvironment (TME) mapping utilizes spatial transcriptomics and single-cell profiling to delineate heterogeneous cell populations and suppressive myeloid niches, while quantifying molecular features such as PD-L1 and tumor mutational burden (TMB). Subsequently, the Epigenetic T-cell Exhaustion Layer visualizes the TF (e.g., TOX/NR4A)-driven transition of functional T cells to a chromatin-closed exhausted state. This informs an Epigenetic Reprogramming Strategy utilizing hypomethylating agents for epigenetic rescue to restore open chromatin and a functional phenotype. The central section details an Allogeneic CAR-T Engineering Platform, where donor-derived T cells undergo CRISPR editing at the genomic level to knock out TRAC (TCR disruption) and B2M (HLA-I removal) to prevent GVHD and evade host NK cells, respectively. Concurrent expression of HLA-E/HLA-G facilitates additional NK cell evasion. This enables a transition from complex ex vivo manufacturing bottlenecks (the ‘VS’ section) toward streamlined, engineered, scalable, off-the-shelf therapies. On the far right, the *In Vivo* CAR-T Generation zone depicts the use of anti-CD8 targeting lipid nanoparticles for mRNA delivery (followed by mRNA translation) or alternative viral vector systems (AAV/lentivirus) to reprogram circulating T cells *in vivo* to generate CAR-Ts. The resulting CAR-T cells exhibit high activity (Enhanced Functional Outcome), including potent tumor killing, tumor regression, and durable response. B2M, beta-2 microglobulin; GVHD, graft-versus-host disease; HLA, human leukocyte antigen; LNPs, lipid nanoparticles; NK, natural killer; PD-L1, programmed death-ligand 1; TMB, tumor mutational burden; TRAC, T-cell receptor alpha constant.

Ultimately, the optimization of CAR-T therapy and BsAbs demands a continuous, reciprocal translation between clinical observations and fundamental immunological mechanisms. The future of oncology lies in the seamless integration of these potent immunotherapeutics into early lines of treatment, informed by deep spatial profiling and facilitated by scalable manufacturing technologies. Identifying epigenetic signatures of terminal exhaustion will allow for the prospective identification of patients destined to fail autologous cellular therapy, representing an emerging biomarker framework that is not yet validated for routine clinical patient stratification (125). Because the absence of human leukocyte antigen class I paradoxically triggers profound natural killer cell-mediated lysis via ‘missing-self’ recognition mechanisms, advanced allogeneic constructs are now being engineered to constitutionally express non-polymorphic human leukocyte antigen-E or human leukocyte antigen-G, though these sophisticated NK evasion strategies remain largely investigational and are confined to early developmental pipelines. By engineering molecular constructs that can actively navigate, resist, and remodel the hostile solid tumor microenvironment, the field aims to systematically overcome established resistance barriers, moving closer toward generating sustainable, durable clinical responses for patients with refractory malignancies (45) (**Figure 9**).
